# A Strategic Approach to Succeed on Clinical Case-Based Multiple-Choice Exams

**DOI:** 10.12688/mep.20542.1

**Published:** 2024-09-20

**Authors:** Animesh Jain, Kunal P. Patel, Gita Fleischman, Neva Howard, Kelly Lacy Smith, Meredith Niess, Erin Bakal, Christina L. Shenvi

**Affiliations:** 1 The University of North Carolina at Chapel Hill School of Medicine, Chapel Hill, North Carolina, USA; 2 WakeMed Raleigh Campus, Raleigh, North Carolina, USA; 3 University of Colorado Anschutz Medical Campus School of Medicine, Aurora, Colorado, USA

**Keywords:** Medical education, Medical students, Undergraduate medical education, Graduate medical education, Educational measurement, Meta-cognitive strategies

## Abstract

Despite the importance of case-based multiple-choice question (CBMCQ) exams, medical educators rarely discuss strategies to systematically approach these questions, and literature on the topic is limited. Through trial-and-error, many students discover or develop more refined and efficient approaches to answering CBMCQs that help them maximize the application of their knowledge base. In this article we present a structured approach to answering CBMCQs, including meta-cognitive strategies to understand the reasons for errors. We provide strategies for success on CBMCQ-based exams and approaches to challenging question types. We also present tips for helping neurodiverse students and an approach for medical educators to teach these skills to their learners.

## Introduction

Case-based multiple-choice questions (CBMCQs) continue to be a core component of evaluating medical learners. High-stakes exams in both undergraduate and graduate medical education, including United States Medical Licensing Examination (USMLE) step exams, National Board of Medical Examiners (NBME) subject exams, and specialty-specific written board exams rely exclusively on CBMCQs. The literature on answering multiple choice questions is sparse. The few reports that do exist describe the problem-solving techniques students use to answer multiple choice questions but do not provide concrete steps to approach questions or improve performance. ( Kolomitro *et al.*, 2020; Prevost & Lemons, 2016). In the medical education arena, there exists a limited body of research that describes predictors of success on national standardized exams, successful study strategies, and specific content resources ( Bin Abdulrahman *et al.*, 2021; Guerrasio *et al.*, 2017; Jackson *et al.*, 2019; Jeyaraju *et al.*, 2023; Khalil *et al.*, 2018). However, we found no literature describing effective strategies to approach CBMCQs.

This absence of literature fits with our own experience whereby medical educators routinely focus on teaching content knowledge, but rarely teach skills specific to answering CBMCQs. Poorly refined test-taking skills can prevent students from fully displaying the knowledge they have worked hard to acquire. Test-taking skills may also be an aspect of the hidden curriculum in medical education and may disproportionately disadvantage certain students.

We propose that meta-cognitive strategies can be taught to help improve performance on CBMCQs. The following tips can be used as a structured approach to help learners systematically and successfully work through CBMCQs, to improve their performance on high-stakes exams. In this article, we presume that the learner already possesses good content knowledge, and we hence focus on test-taking strategies for standardized exams comprised of CBMCQs. Notably, we present an approach that our team of academic coaches and learning specialists have found to often be effective with students based on cumulative 50 decades of experience working with thousands of students. We propose this as one viable approach. Certainly, students and educators may find alternative effective approaches. A key point is that using meta-cognitive reflection and developing a structured approach to questions can be beneficial.

## A six-step approach to CBMCQ’s

**Figure 1.**
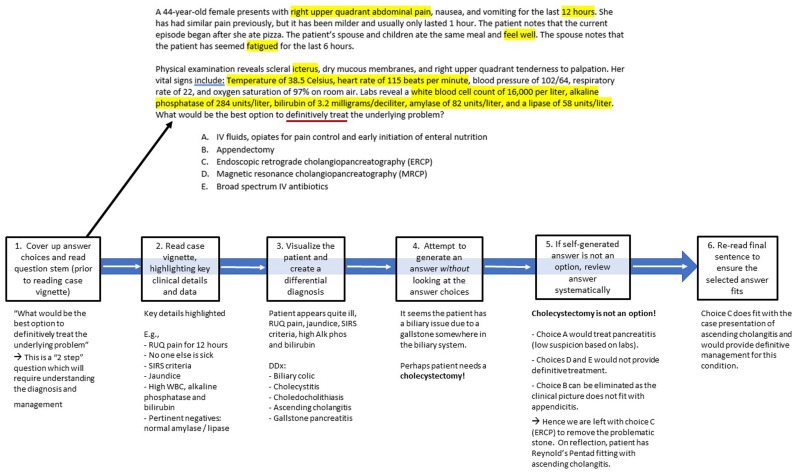
Example case-based multiple-choice question (CBMCQ) and illustration of a structured process to answer the question.

We recommend a standardized approach to all questions. By having a standard approach that is practiced during test-preparation and deployed during test-taking, students can feel less apprehension when facing a high-stakes exam. Here we provide an example question and explain the approach. Figure 1 presents an example of working through a CBMCQ using these 6 steps.

EXAMPLE QUESTION:

A 44-year-old female presents with right upper quadrant abdominal pain, nausea, and vomiting for the last 12 hours. She has had similar pain previously, but it has been milder and usually only lasted 1 hour. The patient notes that the current episode began after she ate pizza. The patient’s spouse and children ate the same meal and feel well. The spouse notes that the patient has seemed fatigued for the last 6 hours.
Physical examination reveals scleral icterus, dry mucous membranes, and right upper quadrant tenderness to palpation. Her vital signs include: temperature of 38.5 Celsius, heart rate of 115 beats per minute, blood pressure of 102/64 mmHg, respiratory rate of 22 per minute, and oxygen saturation of 97% on room air. Labs reveal a white blood cell count of 16,000 per liter, alkaline phosphatase of 284 units per liter, bilirubin of 3.2 milligrams per deciliter, amylase of 82 units per liter, and a lipase of 58 units per liter. What would be the best option to definitively treat the underlying problem?
  A. IV fluids, opiates for pain control and early initiation of enteral nutrition
  B. Appendectomy
  C. Endoscopic retrograde cholangiopancreatography (ERCP)
  D. Magnetic resonance cholangiopancreatography (MRCP)
  E. Broad spectrum IV antibiotics

### Step 1 - First, cover the answer choices and read the final sentence of the question

Case-based questions typically present a case vignette followed by a specific question, such as “what is the likely diagnosis?” or “what is the next step in management?” Students should read the final sentence in the question stem first as it frames the entire case and allows the student to read the vignette with an understanding of what may or may not be significant. In a question asking about diagnosis, clinical information such as symptoms, risk factors, physical exam findings, and laboratory values may be critical to answering the question. In contrast, a question asking about management or pathophysiology is a two-step question in which the student typically needs to determine a diagnosis and know additional information. Reading the final sentence first can help students stay focused and reduce frustration with more challenging questions. In some extreme examples, the question is simply asking for factual knowledge such as the gene associated with a disorder named in the question stem, and the case vignette is largely irrelevant to answering the question. In such cases, a student does not need to waste precious time agonizing over clinical details. The example question presented above asks the student to choose the best option to definitively treat the problem. We hence know this is a two-step question where the student will need to determine a diagnosis and a definitive treatment.

Covering the answer choice can help avoid common pitfalls. One pitfall is a sense of panic if one of the answer choices are unfamiliar. Another pitfall is early anchoring to a familiar term. If the student sees that “troponin” is an answer choice and that the case involves an abnormal EKG, the student may prematurely close their thinking without fully considering the case.

### Step 2 - Read the case vignette, picking up key details

Next, the student should read the case vignette and identify key pieces of information. It is important to read the question efficiently and not dwell excessively on physical exam findings or laboratory values. Judicious use of highlighting can be helpful to focus on key clinical information. Building on step 1, knowing what the question is ultimately asking will help filter the needed key information without highlighting every detail, which is both time-consuming and counterproductive. Using the example question presented above, key details would include the acute time course, abdominal pain, jaundice, fever, and lab abnormalities such as leukocytosis, elevated bilirubin and alkaline phosphatase (see Figure 1).

### Step 3 - Visualize the patient and create a differential diagnosis

On high stakes standardized exams, it is easy to become paralyzed by over-analysis of the multiple-choice options. Given that these questions are typically grounded in clinical reality, coaching the student to visualize the patient sitting in front of them can help focus on the clinical scenario and answer the question effectively. Pausing to visualize the patient and asking “what is the next step” can shift the focus away from exam anxiety and back to clinical knowledge. Even exams in pre-clinical phases of medical education rely heavily on the application of medical knowledge within clinical cases. While more challenging for pre-clinical students, the practice of visualizing the patient can still be helpful since much pre-clinical teaching is focused on pathophysiology of clinical diseases. Using the example above, the student may be able to visualize a similar patient they have seen in the hospital. Given the patient’s acute symptoms, pain, fever, jaundice and lab abnormalities, the student could visualize this is an acutely ill patient who needs urgent intervention. They could then tie this with pathophysiology and imagine the mechanism by which a gallstone could cause such an acute and dramatic presentation.

### Step 4 - Try to generate the correct answer without looking at the answer choices

Once the student has read the full question, they should try to determine the correct answer without looking at the answer choices. If they are unable to come up with the correct answer, a second strategy is to come up with a category of answers. For example, the question stem may be asking for a medication that can treat a patient with hypertensive emergency. The student may not know which medications are listed in the answer choice but could come up with “IV medication to reduce blood pressure” as the category of answer they are looking for. Alternatively, they may be able to come up with several examples of potential answers, such as “an IV beta blocker or IV calcium channel blocker.” Next, they can compare their self-generated answer category to the multiple-choice options. If the answer is in the multiple-choice options, this is highly likely to be the correct answer and the student should confidently move on to the next question. In this situation, the student can briefly skim the other options to make sure they have not made an error, but it is not productive to dwell on the other options or “second guess” the other answer choices. Dwelling can allow the student to convince themselves of an incorrect distractor choice.

### Step 5 - If the self-generated answer is not in the list, work through choices systematically

On many questions, the self-generated answer or answer category may not be one of the multiple-choice options. Here the student can work through the case systematically. First the student can read the multiple-choice options carefully and there may be a “Eureka” moment. If there is still uncertainty, it would be appropriate to briefly re-read the case vignette looking for key details that may differentiate the answer options. If re-reading the question does not yield insights about the correct answer, the student should eliminate any obviously incorrect answers. It would be appropriate here to make an educated guess amongst the remaining choices and move on. Students are often tempted to re-read the question repeatedly looking for a critical piece of information. However, there are diminishing returns from such an approach, and it wastes valuable time on already pressured exams. One meta-cognitive strategy that students can use in this situation is to ask themselves whether more time would help them elucidate the answer. If more time would help, then they can spend a few more moments on it, or flag it to return to later. If more time would not help them figure out the correct answer, they should pick their best guess and move on to save time for questions for which extra time would help them. As educators, we can remind students that there is no penalty for incorrect answers on most standardized exams. Narrowing down to 2 or 3 choices and guessing is often a good strategy. Students should also be reminded that questions often ask for the best answer, not a perfect one, so some uncertainty is to be expected.

### Step 6 – Re-read the final sentence in the question stem and ensure the selected answer fits

Finally, students should re-read the final sentence in the question stem to check that their chosen answer fits and they have not made any careless errors, such as selecting the wrong bubble, answering about the diagnosis rather than management, or anchoring to an incorrect but familiar option. In the case example presented, the final sentence is “What would be the best option to definitively treat the underlying problem?”. Choice C (endoscopic retrograde cholangiopancreatography or ERCP) fits with the clinical diagnosis of ascending cholangitis and would provide definitive therapy of this condition.

## Strategies and approach to challenging questions

In addition to a standardized approach to selecting an answer, students can apply the following five strategies to help maximize the number of correct answers.

### Strategy 1 - Avoid changing answers without a clear reason

When working through questions, it is tempting to change answers especially if there is any uncertainty about the correct answer. This often happens at the end when students are returning to flagged questions and may make a hasty or rushed decision to change answer choices that they are not 100% certain about. However, changing responses often does not yield significant score improvements and can lead to lower scores. When students are considering changing an answer choice, we coach them to ask “is there conclusive evidence from the case that a different answer choice is better than my current best answer?” Students may be tempted to change an answer any time there is uncertainty. Asking the question above forces the student to be analytical and consider if an alternative answer truly fits better. This also helps the student build some tolerance for uncertainty, which is an important skill both on high stakes exams and in clinical patient care.

Students with high levels of test-taking anxiety may be particularly susceptible to second-guessing and changing answers, and these students may already have substantial time pressure. Flagging the question may allow the student to mentally move on and focus on the next question. If there is remaining time, the student can review the flagged questions. However, we encourage students to not change their answer on these flagged questions unless there is a convincing reason that a different answer choice is correct. We also encourage students not to flag all questions where there is doubt, but rather just those questions for which additional time will be helpful. Flagging too many questions may exacerbate anxiety and self-doubt.

One metacognitive strategy that students can use to help develop awareness of how they tend to change answers is to review practice tests and consider how many questions they changed from correct to incorrect, incorrect to correct, or incorrect to incorrect. Often the training software will provide a report of this information. By reviewing their trends, they can determine which questions they tend to change from correct to incorrect answers. It may be a certain content area in which they are less confident. It may be questions that they are rapidly reviewing near the end and when they are feeling rushed. Or there may be other trends that emerge that can help give students greater awareness of when to avoid changing answers without clear, convincing reasons.

### Strategy 2 - Focus on pacing and timing

Many students face significant time pressure on standardized exams, which can be compounded by test-taking anxiety and dwelling on questions. Students can benefit from a clear pacing strategy during the exam. For example, on USMLE Step exams students are provided enough time to average 90 seconds per question. The student can therefore develop guideposts to help with pacing. The student will know that they should have completed about 10 questions by 15 minutes, 20 questions by 30 minutes, and so forth. If the student is behind this pace at each timepoint, they can accelerate their pace dynamically rather than running out of time and missing many questions at the end. It is helpful for students to be reminded that each question is worth the same point value, so dwelling on any one question and therefore rushing or leaving questions unanswered at the end is counterproductive. Importantly, timing strategies should be incorporated into exam preparation. Practice questions should be done in timed mode to simulate the pacing of the actual exam. As students progress in their exam preparation, they should do longer blocks of practice questions to build stamina for the actual exam.

### Strategy 3 – Approach to long question stems, extensive clinical data, or numerous answer choices

Some questions can be challenging due to long case vignettes, extensive clinical data to review such as labs, imaging or EKGs, or numerous answer choices. The common theme in these scenarios is that they can overwhelm and distract a test taker. For these types of questions, it is even more important to have a standardized approach and methodically apply the six tips presented previously. Focusing on the final sentence in the stem (Step 1) can help focus the mind through the long vignette and filter the excessive clinical data. Another approach is to re-word or summarize what the question is asking. In the example question presented above, the case may be summarized as “a woman with right upper quadrant pain, fever, elevated liver enzymes and elevated bilirubin.” By summarizing and removing the extraneous material, the student can focus on the critical aspects of the case and reach the correct response. 

Imaging and histology offer unique challenges because image and histology interpretation are skills that the student may not feel as confident in. However, imaging and histology are often complementary to the other information in the vignette. In many cases, the correct answer may be largely reached primarily from the clinical data, while the imaging or histology serve as confirmation. Alternatively, some cases may hinge on the details of the histology or imaging. In such situations, the histology or imaging is often relatively clear cut and may have been a point of emphasis during the curriculum.

In questions with numerous answer choices, it is critical to not feel overwhelmed but to trust the process of approaching the question using the tips above. Often there will be small differences amongst many of the choices, so noticing a pattern can lead to the elimination of several options. For example, if a question asks about antibiotic options, there may be several redundant options from the same class of antibiotics. By knowing that a particular class is incorrect, multiple options can be eliminated. If the student is not able to eliminate choices or is still overwhelmed, these are excellent questions to flag and move on. Again, we remind students that the worst outcome on a single question is to use excessive time on it and that there is no penalty for guessing.

### Strategy 4 – Approach to very difficult questions

Every examination will have a small percentage of questions that test higher-order and multi-logical thinking. Often, these will present with stems that contain unfamiliar themes or answer choices that require a high level of discrimination. Maintaining a positive mindset when dealing with these types of test questions is paramount. Standardized CBMCQs are almost always norm-referenced exams with some very difficult questions to help create a Gaussian distribution of scores. Test-takers should expect that every exam will have some very difficult questions and know it is not necessary to get all questions correct to receive a good score. It also helps to know that high-stakes exams routinely have a subset of “unscored” or “pilot” questions that do not count towards the actual score but are used to obtain performance statistics prior to actual use.

The following are four strategies for very difficult questions:

1) Be aware of the time (see strategy 2) and set a time-limit to answer this question. Once over the limit, the student should make their best guess, and move on. Excessive vacillation or “analysis paralysis” over an answer choice only wastes more time.
2) If guessing, the student should generally avoid answer options with absolutes such as “always” and “never”. Another high yield clue is if two answers are opposites, one is often, though not always, correct.
3) CBMCQs typically ask for the “single best” answer choice. While multiple answer choices may be partially correct, the student’s task is to choose the best choice. The case vignette will likely contain clues about why one choice is the best option.
4) To maintain a positive mindset while moving to the next question, the student can consider that this question was likely an experimental or unscored question.

### Strategy 5 – Mentally let go of questions that were challenging or required guessing

Often, when a student struggles with a question, or feel they did not know the right answer, it can cause them to feel flustered, discouraged, or more anxious. This negative mental state can lead the student to second-guess themselves on subsequent questions and spiral into worsening anxiety. We encourage students to develop a mental process to recover quickly from questions that have thrown them off. The student may, for example, pick a positive mantra that they say to themselves as they move on from a question that they think they did not answer correctly. Using a “moving on mantra” can create cognitive closure so that the negative emotions from the difficult question do not create a self-doubt spiral. Alternatively, the student could pick a physical act that they repeat in these situations, such as taking a deep breath or tapping their fingers together, that physically and mentally signal that they are letting go of the question and leaving the fear of getting it wrong behind them. This habit can be practiced during training, and applied during actual exams and can help students maintain greater mental clarity.

## Supporting students with neurodiversity

Neurodiverse students, such as those with attention deficit hyperactivity disorder (ADHD) or undiagnosed dyslexia, face unique challenges while taking time-based exams such as the USMLE. Greater difficulty with time awareness and management, difficulty identifying key information in question stems, the risk of making careless errors, mental fatigue, distractibility, and heightened anxiety in test-taking situations may lead to lower scores. Using time management strategies described above (strategy 2) can be particularly important in these situations. It can also be helpful for students to build in centering exercises regularly throughout the exam to maintain focus. For example, we will often advise students to look away from the screen and perform a brief breathing exercise after every 10 or 20 questions to maintain focus.

For students with neurodiversity, it can also be useful to seek multidisciplinary support and testing accommodations. As educators, we may be able to connect students with psychiatry and other specialized services to further develop their executive skills. Most large universities have a testing accommodation office that can provide test modifications, such as extended time, for students with confirmed neurodivergent diagnoses. Moreover, most large testing bodies, such as the USMLE, have a formal process to apply for testing accommodations. Though, in our experience, the process is lengthy, and students may not be granted accommodations despite a formal diagnosis. Importantly, we recommend using the same timing for practice questions and practice exams as what will be used on the exam itself.

## Medical educators can teach a structured approach to answering CBMCQs

The above approach can be taught to students preparing for exams with CBMCQs. The approach to questions can be taught in a large group setting, demonstrating how to cover up the answer choices, read the final sentence, review the vignette identifying the most important pieces of information, and determine the correct answer choice before revealing the options. However, beyond that, each student will have different strengths and challenges. Hence, it is essential for the medical educator to understand each student’s unique struggles and skills. We recommend a structured four step approach to refine test-taking skills with individual students.

### Step 1 – Observe the student completing questions

First the educator should observe the student working through approximately 5 CBMCQ’s on their own. Ask the student to verbalize their thought process aloud as they work through each question. The educator should simply listen and observe without interjecting any suggestions or tips.

### Step 2 – Help elucidate the main challenges

Second, the educator can now determine where the student is struggling. In some cases, a student may lack the baseline medical knowledge to answer the question. However, in other cases, the student may have the knowledge but need coaching in their approach to questions. For example, a student may be glossing over critical physical exam findings, not thinking through a differential diagnosis, or anchoring too early to an answer. Based on these reflections, the educator can provide suggestions for the student using the tips in this article. In particularly difficult situations, the educator can use a microanalytic approach to diagnose the student’s test taking struggles as described by Andrews and colleagues (2018). The goal in this exercise is to help the student develop a greater awareness of their own thought processes during test-taking, and to develop the meta-cognitive strategies outlined above to improve their test-taking capabilities.

### Step 3 – Model test-taking skills

Third the educator can model good test-taking skills by doing 3–5 questions while verbalizing their thought process for each question. The educator can emphasize areas that were challenging for the student. They can demonstrate the standardized approach to questions and use strategies such as determining a category, if they are unsure of the exact answer, of eliminating answer choices if they are unsure of the correct response, or of assessing whether more time would help and moving on with a best guess if not. Importantly, the educator does not have to get all the questions correct to effectively demonstrate the process.

### Step 4 – Allow the student to practice new strategies

Finally, the educator can ask the student to practice these skills by working through 5 new questions and incorporating the suggestions provided. This four-step process may need to be repeated periodically with the student, both as a refresher and to uncover additional challenges. In our experience, this process typically takes 45 minutes. The meeting can be conducted in person or via an online video-conferencing platform where the student shares their screen as they work through the questions.

## Conclusion

Clinical vignette or case-based multiple-choice exams remain the hallmark of assessment in medicine. These exams can be challenging and anxiety-provoking for learners. Here, we present a structured method for approaching case-based multiple-choice questions and meta-cognitive strategies to deal with unique challenges on exams. Furthermore, we describe a process for how we as medical educators can teach these strategies to our learners to help them effectively demonstrate their knowledge and improve their performance.

## Ethics and consent

Ethical approval and consent were not required.

## Data Availability

No data are associated with this article.

## References

[ref-1] Andrews MA Kelly WF DeZee KJ : Why does this learner perform poorly on tests? Using self-Regulated Learning theory to diagnose the problem and implement solutions. *Acad Med.* 2018;93(4):612–615. 10.1097/ACM.0000000000001422 30248085

[ref-2] Bin Abdulrahman KA Khalaf AM Bin Abbas FB : Study habits of highly effective medical students. *Adv Med Educ Pract.* 2021;12:627–633. 10.2147/AMEP.S309535 34135654 PMC8197661

[ref-3] Guerrasio J Nogar C Rustici M : Study skills and test taking strategies for coaching medical learners based on identified areas of struggle. *MedEdPORTAL.* 2017;13: 10593. 10.15766/mep_2374-8265.10593 30800795 PMC6338173

[ref-4] Jackson F Duane E Harmon R : Resources that improve Medical Board Licensing examination performance. *Cureus.* 2019;11(10): e5927. 10.7759/cureus.5927 31788384 PMC6857833

[ref-5] Jeyaraju M Linford H Mendes TB : Factors leading to successful performance on U.S. National Licensure Exams for medical students: a scoping review. *Acad Med.* 2023;98(1):136–148. 10.1097/ACM.0000000000004877 35857389

[ref-6] Khalil MK Williams SE Gregory Hawkins H : Learning and study strategies correlate with medical students' performance in anatomical sciences. *Anat Sci Educ.* 2018;11(3):236–242. 10.1002/ase.1742 28940743

[ref-7] Kolomitro K MacKenzie LW Lockridge M : Problem-solving strategies used in anatomical Multiple-Choice Questions. *Health Sci Rep.* 2020;3(4): e209. 10.1002/hsr2.209 33305012 PMC7714269

[ref-8] Prevost LB Lemons PP : Step by step: biology undergraduates' problem-solving procedures during multiple-choice assessment. *CBE Life Sci Educ.* 2016;15(4): ar71. 10.1187/cbe.15-12-0255 27909021 PMC5132368

