# Supplementary material for: A Strategic Approach to Succeed on Clinical Case-Based Multiple-Choice Exams
Source: MedEdPublish (2016). 2024 Dec 24;14:156. Originally published 2024 Sep 20. [Version 2] doi: 10.12688/mep.20542.2 (PMC11809150; doi:10.12688/mep.20542.2)
Supplement: Peer review history

# Reviewer report: Reviewer response for version 2

**DOI:** 10.21956/mep.22330.r40720

**Published:** 2025-01-17

**Authors:** Janet Mee

**Affiliations:** 1 National Board of Medical Examiners, Philadelphia, Pennsylvania, USA

In this manuscript, the authors propose a methodology for responding to multiple-choice questions that contain a clinical vignette. This topic is worthwhile because CBMCQ is the primary format for many high-stakes assessments in medical education. 

**A few aspects of this article concerned me:**

 1. The authors mention a licensing exam (USMLE) and NBME subject exams but do not reference the materials. USMLE Step 2 CK provides strategies for answering questions (https://www.usmle.org/exam-resources/step-2-ck-materials/step-2-ck-test-question-formats)

 2. The authors state that most high-stakes exams are norm referenced.  This is not the case in medical education. USMLE, Comlex, and NBME subject exams are not norm referenced.  

 3, The proposed framework sounds reasonable, but how do you know it is effective? The authors mention experiences with the process but offer no quantitative support (e.g., students using this process increased their percent correct by x) or qualitative (e.g., X% of students reported feeling more confident . . .)

If evidence from practice is presented, are all the underlying source data available to ensure full reproducibility?

Not applicable

Is the topic of the practical tips discussed accurately in the context of the current literature

Partly

Are all factual statements correct and adequately supported by citations?

Partly

Are the conclusions drawn balanced and justified on the basis of the presented arguments?

No

Are arguments sufficiently supported by evidence from the published literature and/or the authors’ practice?

Yes

Reviewer Expertise:

Educational measurement, standard setting, high-stakes assessment

I confirm that I have read this submission and believe that I have an appropriate level of expertise to state that I do not consider it to be of an acceptable scientific standard, for reasons outlined above.

## References

[1] Step 2 CK Test Question Formats. Reference source

# Reviewer report: Reviewer response for version 2

**DOI:** 10.21956/mep.22330.r40679

**Published:** 2025-01-09

**Authors:** Andrew Cale

**Affiliations:** 1 Indiana University School of Medicine, Indiana, USA

I have no further comments.

If evidence from practice is presented, are all the underlying source data available to ensure full reproducibility?

Not applicable

Is the topic of the practical tips discussed accurately in the context of the current literature

Yes

Are all factual statements correct and adequately supported by citations?

Partly

Are the conclusions drawn balanced and justified on the basis of the presented arguments?

Partly

Are arguments sufficiently supported by evidence from the published literature and/or the authors’ practice?

Partly

Reviewer Expertise:

Metacognition, anatomical sciences, medical education

I confirm that I have read this submission and believe that I have an appropriate level of expertise to confirm that it is of an acceptable scientific standard.

# Reviewer report: Reviewer response for version 2

**DOI:** 10.21956/mep.22330.r40680

**Published:** 2025-01-04

**Authors:** Babu Noushad

**Affiliations:** 1 School of Health Professions Education (SHE), Maastricht University, Maastricht, Netherlands Antilles; 2 College of Health Sciences, University of Buraimi, Buraimi, Oman

The introduction of Dual Process Theory as a framework for explaining the decision-making process while answering CBMCQs has provided structure to the arguments. No further comments.

If evidence from practice is presented, are all the underlying source data available to ensure full reproducibility?

Not applicable

Is the topic of the practical tips discussed accurately in the context of the current literature

Partly

Are all factual statements correct and adequately supported by citations?

Partly

Are the conclusions drawn balanced and justified on the basis of the presented arguments?

Partly

Are arguments sufficiently supported by evidence from the published literature and/or the authors’ practice?

Partly

Reviewer Expertise:

Metacognitive monitoring, Instructional designs, Cognitive psychology

I confirm that I have read this submission and believe that I have an appropriate level of expertise to confirm that it is of an acceptable scientific standard.

# Reviewer report: Reviewer response for version 1

**DOI:** 10.21956/mep.21987.r38950

**Published:** 2024-10-02

**Authors:** Andrew Cale

**Affiliations:** 1 Indiana University School of Medicine, Indiana, USA

In this article the authors described a strategic method of approaching clinical case-based multiple choice questions based largely on their collective experience as educators who have worked with thousands of students. The authors include an initial six-step approach as well as strategies for overcoming challenging questions and supporting neurodiverse students.

 Overall, I believe this article provides useful strategies for both students and educators on how to approach these challenging case-based MCQs. However, I would like to see additional references that also evidentially support the strategies listed.

 Additionally, the authors mention that these strategies can help support the students' metacognition. While I believe this is true, it would be helpful if the authors directly linked these strategies to metacognitive principles more explicitly and supported these claims with references to the metacognition literature.

If evidence from practice is presented, are all the underlying source data available to ensure full reproducibility?

Not applicable

Is the topic of the practical tips discussed accurately in the context of the current literature

Yes

Are all factual statements correct and adequately supported by citations?

Partly

Are the conclusions drawn balanced and justified on the basis of the presented arguments?

Partly

Are arguments sufficiently supported by evidence from the published literature and/or the authors’ practice?

Partly

Reviewer Expertise:

Metacognition, anatomical sciences, medical education

I confirm that I have read this submission and believe that I have an appropriate level of expertise to confirm that it is of an acceptable scientific standard, however I have significant reservations, as outlined above.

# Reviewer report: Reviewer response for version 1

**DOI:** 10.21956/mep.21987.r38943

**Published:** 2024-09-25

**Authors:** Babu Noushad

**Affiliations:** 1 School of Health Professions Education (SHE), Maastricht University, Maastricht, Netherlands Antilles; 2 College of Health Sciences, University of Buraimi, Buraimi, Oman

This article aims to provide several tips for effectively solving clinical case-based MCQ exams. The authors have identified key areas for educators and learners to focus on. However, the manuscript has room for significant improvement, and a few suggestions are provided below to enhance it further. 

 1. The manuscript presents 13 tips under three headings (a six-step approach, strategies for challenging questions, and tips to modify instructional approaches), which makes the content less targeted to a specific audience (learners or educators). The authors may want to consider restructuring the manuscript as a twelve-tips article, a more familiar format in this journal for presenting practical tips. Additionally, it would be more effective to focus these tips on a single audience. A recommended approach is to direct the tips specifically toward faculty.

 2. The manuscript lacks a robust theoretical framework, which weakens the evidence base of the study. As a result, the arguments presented are primarily subjective, drawing mostly from the author's personal experiences and perceptions. To strengthen the manuscript, the authors may consider incorporating the 'Dual Processing Theory of Human Decision Making' (System-1 and System-2), as well as Zimmerman's Model of Self-Regulated Learning. This will provide a solid framework, making the arguments and line of reasoning more evidence-based and valid. 

 3. The correct term is **"metacognitive"** (without the hyphen).

If evidence from practice is presented, are all the underlying source data available to ensure full reproducibility?

Not applicable

Is the topic of the practical tips discussed accurately in the context of the current literature

Partly

Are all factual statements correct and adequately supported by citations?

Partly

Are the conclusions drawn balanced and justified on the basis of the presented arguments?

Partly

Are arguments sufficiently supported by evidence from the published literature and/or the authors’ practice?

Partly

Reviewer Expertise:

Metacognitive monitoring, Instructional designs, Cognitive psychology

I confirm that I have read this submission and believe that I have an appropriate level of expertise to confirm that it is of an acceptable scientific standard, however I have significant reservations, as outlined above.

## References

[1] : Dual processing model of medical decision-making. *BMC Med Inform Decis Mak* .2012;12: 10.1186/1472-6947-12-94 94 10.1186/1472-6947-12-94 22943520 PMC3471048

[2] : Dual-Process Theories of Higher Cognition: Advancing the Debate. *Perspect Psychol Sci* .2013;8(3) : 10.1177/1745691612460685 223-41 10.1177/1745691612460685 26172965
